# RIPK1 Coordinates Bone Marrow Mesenchymal Stem Cell Survival by Maintaining Mitochondrial Homeostasis via p53

**DOI:** 10.1155/2021/5540149

**Published:** 2021-11-19

**Authors:** Qing Tian, Chen Cao, Weijian Qiu, Han Wu, Lijun Zhou, Zhipeng Dai, Zhenwei Li, Songfeng Chen

**Affiliations:** ^1^Department of Orthopaedics, The First Affiliated Hospital of Zhengzhou University, Zhengzhou 450052, China; ^2^Department of Orthopaedics, Henan Provincial People's Hospital, Zhengzhou 450003, China; ^3^Department of Orthopaedics, Union Hospital, Tongji Medical College, Huazhong University of Science and Technology, Wuhan 430022, China

## Abstract

Survival of mesenchymal stem cells in the bone marrow is essential for bone marrow microenvironment homeostasis, but the molecular mechanisms remain poorly understood. RIPK1 has emerged as a critical molecule of programmed cell death in tissue homeostasis. However, little is known about the regulation of RIPK1 on bone marrow mesenchymal stem cells (MSCs). Here, we have investigated for the first time the role of RIPK1 in bone marrow MSCs. We have found that RIPK1 knockdown suppressed proliferation, differentiation, and migration in bone marrow MSCs. Furthermore, RIPK1 knockdown resulted in the opening of mitochondrial permeability transition pore (mPTP) and mtDNA damage, leading to mitochondrial dysfunction, and consequently induced apoptosis and necroptosis in bone marrow MSCs. Moreover, we identified that the p53-PUMA axis pathway was involved in mitochondrial dysfunction in RIPK1-deficient bone marrow MSCs. Together, our findings highlighted that RIPK1 was indispensable for bone marrow MSC survival.

## 1. Introduction

Bone marrow microenvironment plays a critical role for maintenance of bone homeostasis [1–3]. Moreover, the dysregulation of bone marrow microenvironment has been implicated in many human diseases, including osteoporosis, osteonecrosis of the femoral head (ONFH), myelodysplastic syndrome (MDS), and multiple myeloma (MM) [4, 5]. Mesenchymal stem cells (MSCs) residing in the bone marrow are a key component of the microenvironment and exhibit multipotency and self-renewal capabilities [6–8]. MSCs maintain and regulate the homeostasis of bone marrow microenvironment by proliferation and differentiation into multiple cell types [9, 10]. Bone marrow microenvironment is a dynamic system that requires balanced cell survival and death of MSCs [11]. Therefore, it is essential to uncover the molecular mechanisms underlying the fate decisions of MSCs.

Receptor-interacting protein kinase 1 (RIPK1) has been identified as a master regulator of the cellular fate decision in tissue homeostasis and development [12–14]. RIPK1 coordinates two opposed cellular fates: cell survival via NF-*κ*B-mediated gene induction and cell death by induction of apoptosis or necroptosis [15–17]. Recently, an increasing number of findings have highlighted that RIPK1 is critical for the fate determinations of stem cells in tissue regeneration and development [18–20]. RIPK1-deficient hematopoietic stem cells (HSCs) failed to self-renew and differentiate into megakaryocyte-erythroid progenitors and granulocyte-macrophage progenitors [20]. Meanwhile, conditional deletion of RIPK1 in mice directly results in increased hematopoietic stem cell death and subsequent bone marrow failure, whereas mice with kinase-inactive RIPK1 can develop normally [18]. However, it is unclear whether RIPK1 is essential for bone marrow MSC fate decisions.

In the current study, we first explored the function and mechanisms of RIPK1 on bone marrow MSCs in vitro. We have found that RIPK1 knockdown suppressed proliferation, differentiation, and migration in bone marrow MSCs. Furthermore, RIPK1 knockdown resulted in the opening of mPTP (mitochondrial permeability transition pore) and mtDNA (mitochondrial DNA) damage, leading to mitochondrial dysfunction, and consequently induced apoptosis and necroptosis in bone marrow MSCs. Moreover, we identified that the p53-PUMA axis pathway was involved in mitochondrial dysfunction in RIPK1-deficient bone marrow MSCs. These findings highlighted that RIPK1 was indispensable for bone marrow MSC survival.

## 2. Materials and Methods

### 2.1. Isolation and Culture of Primary Bone Marrow MSCs

All experimental procedures were approved by the animal experimentation committee of Zhengzhou University. The male Sprague-Dawley rats (6 weeks old) were purchased from the Experimental Animal Center of Zhengzhou University. The isolation and culture methods of bone marrow MSCs were performed as previously described [21]. The second-generation bone marrow MSCs were used in the following experiments.

### 2.2. Transfection and siRNA Knockdown

Rat siRIPK1 and sip53 were designed and synthesized by Ribo Biological Technology (Ribo, Guangzhou, China) according to previously guidelines [22]. The sequences of siRIPK1 are as follows: 5′-GGATAATCGTGGAGATCATdTdT-3′, 5′-AUGAUCUCCACGAUUAUCCdTdT-3′; 5′-GUCUUCGCUAACACCACUAdTdT-3′, 5′-UAGUGGUGUUAGCGAAGACdTdT-3′; 5′-GGAACAACGGAGTATATAAdTdT-3′, 5′-UUAUAUGUUGUUCCdTdT-3′; and sip53: 5′-UCAAAUCAUCCAUUGCUTTTdTdT-3′, 5′-AAAACCUUAAAAUCUAATTdTdT-3′; UACAUUAUUUCAUUAAATTdTdT-3′, 5′-AAAACCUUAAAAACAUTTdTdT-3′; 5′-UUACACCUUCAAACAUTdT dT-3′, 5′-UUACAUCACCCUACCAUCTdTdT-3′, respectively. The negative control group was transfected with nonspecific siRNA. The primary bone marrow MSCs were transfected with siRNA sequence at a concentration of 100 pmol/10^5^ cells with Lipofectamine RNAiMAX (Invitrogen). After 24 h of transfection, the knockdown efficiency of siRNA was evaluated by western blotting. Then, the effective siRNA sequence was selected and applied for subsequent experiments.

### 2.3. Cell Proliferation and Viability Assay

After transfection with siRNA, bone marrow MSCs were collected and seeded into the 96-well plate at a density of 1 × 10^4^ cells/well. Next, the Cell Counting Kit-8 assay (Dojindo Biotechnology, Japan) was used to evaluate cell proliferation according to the manufacturer's instructions [23]. After treatment, the medium was removed and replaced with mixture solution containing 90 *μ*l culture medium and 10 *μ*l CCK-8 solution. Then, after incubation for 4 h at 37°C in the dark, each sample was determined spectrophotometrically at 450 nm.

### 2.4. Hoechst 33258 and EDU Staining

The primary bone marrow MSCs were seeded into 24-well culture plates. Then, the medium was removed and the plates were washed twice with PBS. Subsequently, Hoechst 33258 (10 mg/ml) and EDU (5 mg/ml) were used to stain bone marrow MSCs [24]. The staining was protected from light for 10 min; then, bone marrow MSCs were washed with PBS and observed under a laser confocal scanning microscope (OLYMPUS FV1000, Japan).

### 2.5. Cell Invasion Assay In Vitro

According to the instructions, 1 × 10^4^ cells and 1% FBS were added to each upper chamber, and each lower chamber was filled with FBS for transmembrane induction. After being incubated for 24 h at 37°C, cells on the upper surface of the upper chamber were removed with cotton swabs. The cells invading to the other side of the upper chamber were fixed with methanol for 30 min and stained with 1% crystal violet, then counted and calculated using a microscope [25].

### 2.6. Alizarin Red Staining

To visualize the effect of RIPK1 on osteogenic differentiation, Alizarin Red staining was performed on MSCs. Briefly, after posttransfection, modified MSCs were cultured in osteogenesis differentiation media (Cyagen Biosciences, China). Then, cells were fixed, washed, and stained in 2% Alizarin Red S solution (Sigma-Aldrich) according to the manufacturer's protocol. After incubation for 30 min, the cells were washed with staining buffer and photographed by a microscope (Olympus, Japan) [26].

### 2.7. Oil Red O Staining

To visualize the effect of RIPK1 on adipogenic differentiation, Oil Red O staining was performed on MSCs. Briefly, after posttransfection, modified MSCs were cultured in adipogenic differentiation media (Cyagen Biosciences, China). Then, cells were fixed, washed, and stained in Oil Red O dye solution (Beyotime Biotech, China) according to the manufacturer's protocol. After incubation for 30 min, the cells were washed with staining buffer and photographed by a microscope [27].

### 2.8. Alcian Blue Staining

To visualize the effect of RIPK1 on chondrogenic differentiation, Alcian Blue staining was performed on MSCs. Briefly, after posttransfection, modified MSCs were cultured in chondrogenesis differentiation media (Cyagen Biosciences, China). Then, cells were fixed, washed, and stained in Alcian Blue dye solution (Beyotime Biotech, China) according to the manufacturer's protocol. After incubation for 30 min, the cells were washed with staining buffer and photographed by a microscope [28].

### 2.9. Transmission Electron Microscopy (TEM)

TEM was used to detect cells with apoptotic and necrotic morphology. Briefly, the primary bone marrow MSCs were collected, centrifuged, and rinsed twice with PBS. Samples were then fixed in 2.5% glutaraldehyde for 2 h and postfixed with 1% osmium tetroxide for 2 h at 37°C. Subsequently, cells were dehydrated with ethanol and embedded in Epon-812. Ultrathin sections were prepared by staining with lead citrate and uranyl acetate; thus, ultrastructure changes were able to be evaluated by TEM (FEI Tecnai-20, Holland) [23].

### 2.10. Cell Death Determination by Annexin-V-FITC/Propidium Iodide Staining

After transfection with siRNA, the primary bone marrow MSCs were stained using Annexin-V-FITC/propidium iodide (PI) kit (KeyGen Biotech, China) [29]. Briefly, the primary bone marrow MSCs were harvested and resuspended with 500 *μ*l binding buffer containing 5 *μ*l Annexin-V and 5 *μ*l PI. After incubation according to the manufacturer's instructions, the samples were detected by flow cytometry (Becton Dickinson, NJ, USA).

### 2.11. Mitochondrial Membrane Potential (MMP)

The MMP of primary bone marrow MSCs was examined as previously described [23]. Briefly, the primary bone marrow MSCs were harvested and resuspended with mixture solution containing 0.5 ml fresh medium and 0.5 ml JC-1 (Beyotime Biotech, China) staining solution. After incubation, the samples were washed with staining buffer and detected by flow cytometry.

### 2.12. Mitochondrial Permeability Transition Pore (mPTP)

The mPTP of primary bone marrow MSCs was examined by mPTP Assay Kit (Beyotime Biotech, China) according to the manufacturer's instructions [23]. Finally, the relative fluorescence intensity (RFI) of all samples was detected by flow cytometry.

### 2.13. Analysis of Mitochondrial DNA

To observe of the change of mitochondrial DNA in situ, the primary bone marrow MSCs were prepared by staining with MitoTracker™ Red CMXRos (Invitrogen, Life Technologies, USA) and PicoGreen™ (Invitrogen, Life Technologies, USA) according to the manufacturer's protocol [30]. Briefly, mitochondria and mitochondrial DNA were stained with 100 nM MitoTracker Red CMXRos and 3 *μ*g/ml PicoGreen, respectively. Finally, the primary bone marrow MSC samples were observed under a laser scan confocal microscope.

### 2.14. Detection of Mitochondrial ATP

The mitochondria were isolated according to the manufacturer's protocol of the Mitochondria Isolation Kit (Beyotime Biotech, China) [22]. Next, the mitochondrial ATP content was measured using ATP Assay Kit (Beyotime Biotech, China). The mitochondria were incubated in 200 *μ*l of lysis buffer. The supernatant was collected and quantified by the BCA Protein Assay Kit (Beyotime Biotech, China). Finally, 100 *μ*l detection reagent was added to 100 *μ*l supernatant, and then, the luciferase activity was determined by luminescence spectrometry (Enspire, USA). Relative level of the mitochondrial ATP was expressed as the fold of luciferase activity of treated groups to the control.

### 2.15. Western Blotting

Cells for protein extraction were cultured in appropriate plates. When 80-90% confluence is reached, they were harvested and lysed in the RIPA lysis buffer (Boster Biological Technology, Wuhan, China). After being centrifugated at 12000 × g for 10 min at 4°C, the supernatant was collected and protein was determined by an Enhanced BCA Protein Assay Kit (Beyotime Biological Technology, Shanghai, China). Then, samples were separated by 12% SDS-PAGE and transferred to the nitrocellulose membranes. The membrane was later blocked with blocking buffer for 2 h at room temperature (RT) and incubated with primary antibodies overnight at 4°C. Then, membranes were washed three times with TBST and incubated with horseradish peroxidase-conjugated secondary antibodies for 2 h at RT. Antibodies were detected by the enhanced chemiluminescence kit (Boster Biological Technology, Wuhan, China), and membranes were exposed to a light-sensitive film. The primary antibodies used in this study were as follows: anti-RIPK1 (1 : 500, CST, USA), anti-RIPK3 (1: 1000, Abcam, USA), anti-Phospho-RIPK3 (1 : 1000, Abcam, USA), anti-MLKL (1: 1000, CST, USA), anti-Phospho-MLKL (1 : 1000, CST, USA), anti-Runx2 (1 : 1000, CST, USA), anti-PPAR*γ* (1 : 1000, Abcam, USA), anti-Sox9 (1 : 10000, Abcam, USA), anti-p53 (1 : 1000, Abcam, USA), anti-PUMA (1 : 1000, Abcam, USA), anti-Cleaved caspase-3 (1 : 1000, Abcam, USA), anti-VDAC1 (1 : 500, Abcam, USA), and anti-*β*-Actin (1 : 1000, Abcam, USA).

### 2.16. Statistical Analysis

All experiments were conducted for at least three times, and data were shown as the mean ± standard deviation (SD). Statistical analyses were performed with SPSS 18.0 using one-way analysis of variance (ANOVA) to compare different samples. Student's *t*-tests were also used to compare the differences between two groups, and *p* < 0.05 was considered statistically significant.

## 3. Results

### 3.1. Knockdown of RIPK1 in Bone Marrow MSC Suppresses Cell Proliferation, Differentiation, and Migration

**Figure 1:**
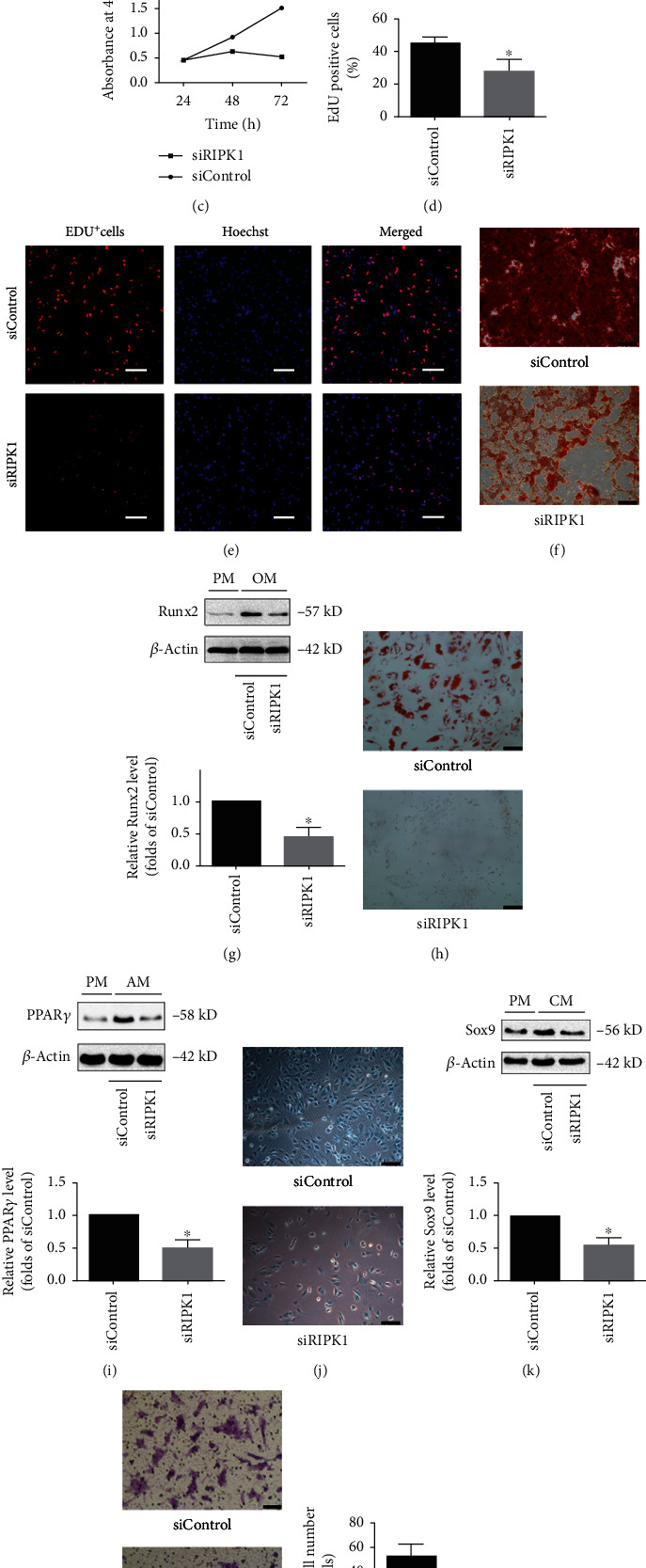
Biological function of RIPK1 on proliferation, differentiation, and migration of bone marrow MSCs. (a) The bone marrow MSCs were transfected with siControl or siRIPK1 for 24 h; then, knockdown efficiency of RIPK1 was assessed by western blotting. Representative western blots of the expression of RIPK1. Histogram analysis showing the relative protein levels of RIPK1. Data are presented as the means ± SD from three independent experiments (^∗^*p* < 0.05, vs. siControl; *n* = 3). siRIPK1(#1) showed the highest efficiency among three siRIPK1. (b) Morphologic changes in bone marrow MSCs after 72 h of siRNA transfection. In the siRIPK1 group, more cells detached from culture plates with a morphology transition (scale bar = 100 *μ*m). (c) Proliferation of bone marrow MSCs was estimated by CCK-8 assay at 24, 48, and 72 h after transfection with siRNA. Knockdown of RIPK1 resulted in inhibition of cell proliferation, compared with the control group. Values are expressed as the means ± SD from three independent experiments (^∗^*p* < 0.05, vs. siControl; *n* = 5). (d) Quantification of EDU-positive cells in two groups, showing that knockdown of RIPK1 led to decrease of EDU incorporation. Values are expressed as the means ± SD from three independent experiments (^∗^*p* < 0.05, vs. siControl; *n* = 3). (e) Representative images of EDU incorporation in bone marrow MSCs, and nuclei were stained with Hoechst (scale bar = 100 *μ*m). (f, g) Effect of RIPK1 on osteogenic differentiation in bone marrow MSCs. The Alizarin Red staining of bone marrow MSCs after incubation with osteogenic medium for 14 d (scale bar = 200 *μ*m) (f). Representative western blots of the expression of Runx2. Histogram analysis showing the relative protein levels of Runx2. Data are presented as the means ± SD from three independent experiments (^∗^*p* < 0.05, vs. siControl; *n* = 3) (PM: proliferative medium; OM: osteogenic medium) (g). (h, i) Effect of RIPK1 on adipogenic differentiation in bone marrow MSCs. The Oil Red O staining of bone marrow MSCs after incubation with adipogenic medium for 14 d (scale bar = 100 *μ*m) (h). Representative western blots of the expression of PPAR*γ*. Quantitative data showing the relative protein levels of PPAR*γ*. Data are presented as the means ± SD from three independent experiments (^∗^*p* < 0.05, vs. siControl; *n* = 3) (PM: proliferative medium; AM: adipogenic medium) (i). (j, k) Effect of RIPK1 on chondrogenic differentiation in bone marrow MSCs. The Alcian Blue staining of bone marrow MSCs after incubation with chondrogenic medium for 14 d (scale bar = 100 *μ*m) (j). Representative western blots of the expression of Sox9. Quantitative data showing the relative protein levels of Sox9. Data are presented as the means ± SD from three independent experiments (^∗^*p* < 0.05, vs. siControl; *n* = 3) (PM: proliferative medium; CM: chondrogenic medium) (k). (l, m) Effect of RIPK1 on migration of bone marrow MSCs. Transwell invasion assay (l) and quantitative data (m) showed significant decrease of invasive cells in siRIPK1 group. Values are expressed as the means ± SD from three independent experiments (^∗^*p* < 0.05, vs. siControl; *n* = 3).

To analyze the function of RIPK1 in bone marrow MSCs, we systematically knocked down RIPK1 with siRNAs. First, we silenced RIPK1 as confirmed by western blot. As shown in Figure 1(a), in comparison to the siRNA control group, the siRIPK1(#1) group showed the highest knockdown efficiency. In the following experiment, we silenced RIPK1 in bone marrow MSCs by transfection with siRIPK1(#1). After transfection with siRIPK1(#1) for 72 h, bone marrow MSCs lost their normal morphology, displayed significant shrinkage, and detached from the plates (Figure 1(b)).

To explore the importance of RIPK1 for the proliferation of bone marrow MSCs, CCK-8 assay was used to evaluate cell proliferation in RIPK1-deficient cells. Intriguingly, we found that cell proliferation was obviously decreased in RIPK1-deficient cells versus control cells (Figure 1(c)). Furthermore, we investigated whether RIPK1 is required for cell proliferation in bone marrow MSCs, using the EDU incorporation assay to detect DNA synthesis during cell growth. Similarly, knockdown of RIPK1 in bone marrow MSCs resulted in the decrease of EDU-positive cells compared with the control cells (Figures 1(d) and 1(e)). These findings implied that RIPK1 was required for cell proliferation in bone marrow MSCs.

We next estimated the effect of RIPK1 knockdown on multilineage differentiation potential, another key characteristic of bone marrow MSCs. To this end, we cultured RIPK1-deficient bone marrow MSCs by osteogenic induction media. Remarkably, knockdown of RIPK1 in bone marrow MSCs reduced osteogenic differentiation as demonstrated by Alizarin Red staining (Figure 1(f)). Consistently, the expression of Runx2, a crucial transcription factor for osteogenic differentiation, was significantly decreased in RIPK1 knockdown cells (Figure 1(g)). These findings suggested that RIPK1 deficiency impaired osteogenic differentiation in bone marrow MSCs.

To explore whether RIPK1 affects adipogenic differentiation in bone marrow MSCs, we used adipogenic induction medium to induce differentiation in cultured RIPK1-deficient bone marrow MSCs. The bone marrow MSCs were stained with Oil Red O stain following incubation with the adipogenic medium. Lipid accumulation was also attenuated in RIPK1-deficient bone marrow MSCs compared with the control group (Figure 1(h)). Consistently, the expression of PPAR*γ*, a crucial transcription factor for adipogenic differentiation, was significantly decreased in RIPK1 knockdown cells (Figure 1(i)). These results suggested that RIPK1 deficiency inhibited adipogenic differentiation.

To explore whether RIPK1 affects chondrogenic differentiation in bone marrow MSCs, we used chondrogenic induction medium to induce differentiation in cultured RIPK1-deficient bone marrow MSCs. As shown in Figure 1(j), knockdown of RIPK1 in bone marrow MSCs reduced chondrogenic differentiation as demonstrated by Alcian Blue staining. Consistently, the expression of Sox9, a crucial transcription factor for chondrogenic differentiation, was significantly decreased in RIPK1 knockdown cells (Figure 1(k)). These results suggest that RIPK1 deficiency inhibited chondrogenic differentiation.

Bone marrow MSC homing is an essential process for maintenance bone homeostasis and hematopoiesis [31]. The homing process involves cell migration, a basic characteristic of bone marrow MSCs. To determine whether RIPK1 deficiency impaired homing of bone marrow MSCs, we evaluated cell migration using transwell invasion assay. As shown in Figures 1(l) and 1(m), compared with the control group, the average invasive cell number of RIPK1-deficient bone marrow MSCs significantly reduced.

Taken together, the above experiments indicated that RIPK1 was indispensable for cell proliferation, differentiation, and migration in bone marrow MSCs.

### 3.2. Characterization of Bone Marrow MSC Cell Death Induced by Knockdown of RIPK1

**Figure 2:**
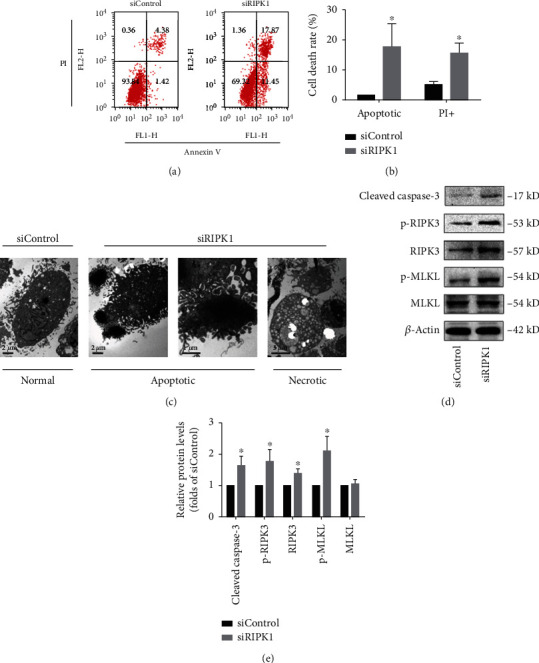
Effect of RIPK1 on cell death of bone marrow MSCs. (a) Representative graphs were analyzed using the flow cytometer. After 72 h transfection with siRNA in bone marrow MSCs, Annexin V/PI staining was used to determine the type of cell death. Annexin V-positive and PI-negative represented apoptotic cells, while PI-positive represented necrotic cells. (b) Histogram analysis illustrated the increase of apoptotic and necrotic cells in RIPK1-deficient bone marrow MSCs. Values are expressed as the means ± SD from three independent experiments (^∗^*p* < 0.05, vs. siControl; *n* = 3). (c) Typical TEM images of morphological ultrastructural changes in bone marrow MSCs. After transfection with siRIPK1 for 72 h, bone marrow MSCs displayed both apoptotic ultrastructural changes including condensed nuclei and apoptotic body and necrotic ultrastructural changes including organelle swelling, plasma membrane disruption, and cellular lysis (scale bar = 1 *μ*m or 2 *μ*m). (d) Representative western blots for the expression of Cleaved caspase-3, p-RIPK3, p-MLKL, and corresponding total protein. (e) Histogram analysis showing the relative protein levels of Cleaved caspase-3, p-RIPK3, p-MLKL, and corresponding total protein. Data are presented as the means ± SD from three independent experiments (^∗^*p* < 0.05, vs. siControl; *n* = 3).

As a master player for cell fate determination, RIPK1 is essential for cell survival and death [32]. To explore the type of cell death in bone marrow MSCs induced by knockdown of RIPK1, cell death was evaluated by Annexin V/PI staining. Annexin V-positive and PI-negative staining bone marrow MSCs represent apoptotic cells. PI-positive staining bone marrow MSCs represent necrotic cells. As shown in Figures 2(a) and 2(b), the percentages of apoptotic and necrotic cells in RIPK1-deficient bone marrow MSCs were obviously increased compared with the control cells. To further confirm that apoptotic and necrotic cell death was induced by knockdown of RIPK1, we also performed TEM to observe morphological changes in bone marrow MSCs. After knockdown of RIPK1, the bone marrow MSCs exhibited apoptotic morphological features (such as cell shrinkage, nuclear condensation, and apoptotic body formation) as well as necrotic morphological features (such as severe vacuolation, organelle swelling, and cellular lysis) (Figure 2(c)).

Necroptosis, one form of programmed necrotic cell death, can be activated under RIPK1-deficient conditions [33]. To investigate whether necrotic cell death induced by RIPK1 deficiency in bone marrow MSCs, western blot analysis was used to evaluated the expression of key regulators of the necroptotic pathway. As shown in Figures 2(d) and 2(e), after knockdown of RIPK1 for 72 h in bone marrow MSCs, we detected upregulation of RIPK3. However, MLKL, the main executor of necroptosis, was unchanged in RIPK1 knockdown bone marrow MSCs. Intriguingly, we found that the phosphorylation levels of RIPK3 and MLKL were all increased in RIPK1-deficient bone marrow MSCs, suggesting that the necroptotic pathway was involved in RIPK1 deficiency-induced necrotic cell death. In addition to necroptosis, Cleaved caspase-3, a molecular marker of apoptosis, was also upregulated in RIPK1-deficient bone marrow MSCs (Figures 2(d) and 2(e)).

Collectively, our results revealed that RIPK1 acted as an inhibitor for apoptotic and necroptotic cell death in bone marrow MSCs, suggesting that RIPK1 was critical for cell survival.

### 3.3. Knockdown of RIPK1 Impaired Mitochondrial Homeostasis in Bone Marrow MSCs

**Figure 3:**
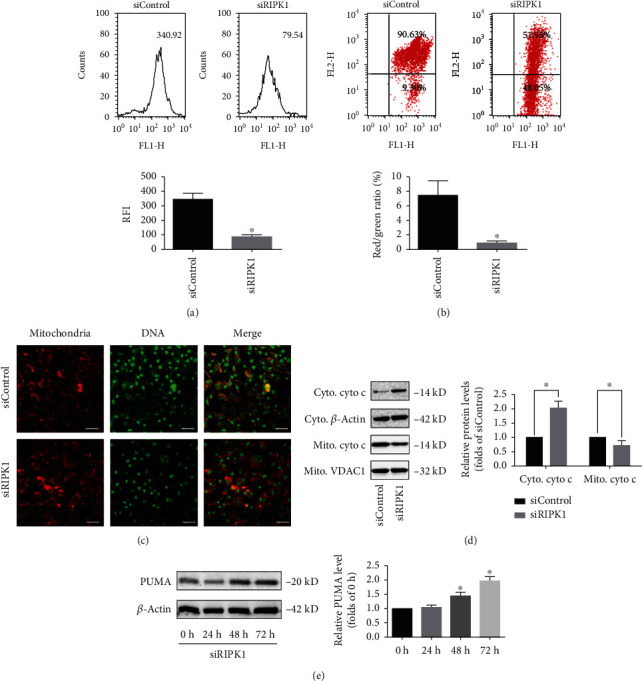
Effect of RIPK1 on mitochondrial homeostasis of bone marrow MSCs. (a) After 72 h transfection with siRNA in bone marrow MSCs, the opening of mPTP was analyzed by the flow cytometer using mPTP Assay Kit. Representative graphs illustrated the increase of mPTP opening after knockdown of RIPK1. The quantitative mPTP in bone marrow MSCs was demonstrated by RFI. Data are expressed as the means ± SD from three independent experiments (^∗^*p* < 0.05, vs. siControl; *n* = 3). (b) Representative graphs illustrated the loss of MMP after knockdown of RIPK1. The quantitative MMP in bone marrow MSCs was demonstrated by red/green ratio. Data are presented as the means ± SD from three independent experiments (^∗^*p* < 0.05, vs. siControl; *n* = 3). (c) Representative confocal images were obtained in situ staining with MitoTracker (mitochondria, red) and PicoGreen (DNA, green). Knockdown of RIPK1 promotes mitochondrial DNA release into the cytoplasm (scale bar = 50 *μ*m). (d) Representative western blots showing the release of cytochrome c. Histogram analysis showing the relative protein levels of cytochrome c. Data are presented as the means ± SD from three independent experiments (^∗^*p* < 0.05, vs. siControl; *n* = 3). (e) Representative western blots of the expression of PUMA. Histogram analysis showing the relative protein levels of PUMA. Data are presented as the means ± SD from three independent experiments (^∗^*p* < 0.05, vs. siControl; *n* = 3).

Mitochondrial homeostasis is essential for the initiation of cell death in bone marrow MSCs [26]. To further investigate the mechanisms underlying RIPK1 deficiency-induced cell death in bone marrow MSCs, we explored its possible effects on mitochondria. The opening of mPTP is recognized as an initial step to activating apoptosis or necroptosis [22, 23]. Once mPTP opens, mtDNA and cytochrome c could release from mitochondria into cytosol via mPTP, which subsequently activated apoptotic cell death pathway, necroptotic cell death pathway, or both. As shown in Figure 3(a), after knockdown of RIPK1 for 72 h, the value of RFI was significantly decreased, suggesting that the opening rate of mPTP in bone marrow MSCs was augmented. The similar data was also observed in MMP, which could be collapse upon mPTP opening (Figure 3(b)). Subsequently, we found that the opening of mPTP contributed to leakage of mtDNA and cytochrome c from mitochondria into cytosol of RIPK1-deficient bone marrow MSCs (Figures 3(c) and 3(d)).

PUMA, a BH3-only Bcl-2 family member, has been identified as a critical regulator for mitochondrial homeostasis. PUMA could bind Bcl-2 to activate Bax and Bak and lead to the opening of mPTP and cell death [34, 35]. Since PUMA is required for the opening of mPTP, we examined the expression of PUMA in RIPK1-deficient bone marrow MSCs. Consistent with the previous study, the expression of PUMA was increased in a time-dependent manner after knockdown of RIPK1 (Figure 3(e)).

### 3.4. p53 Mediates Suppression of Proliferation and Differentiation in RIPK1-Deficient Bone Marrow MSCs

**Figure 4:**
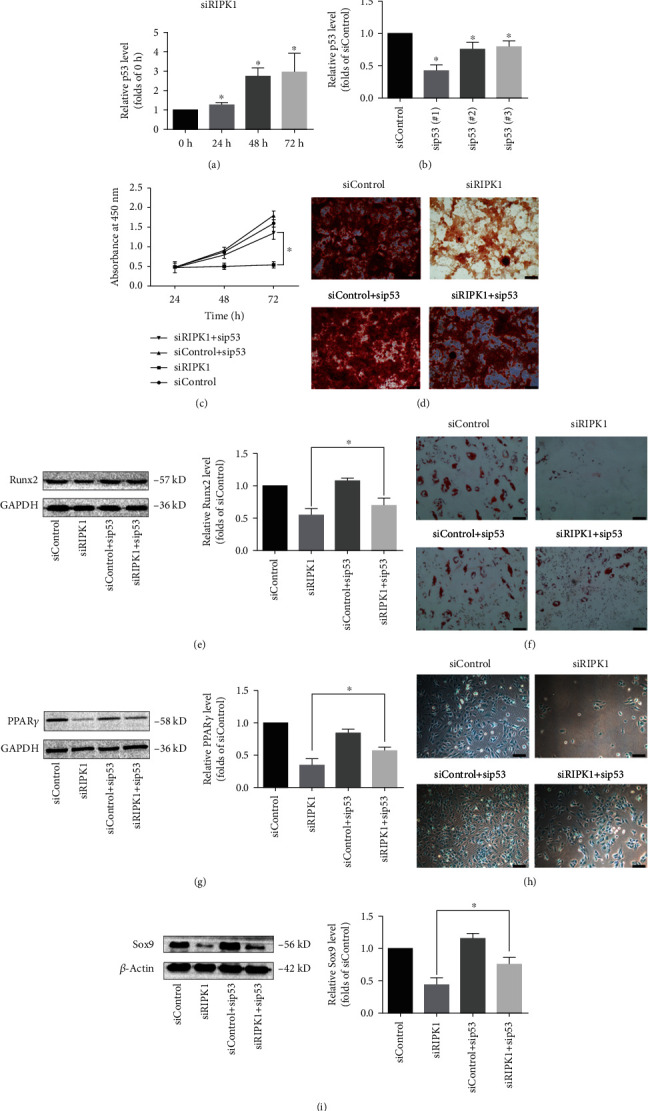
RIPK1 regulated proliferation and differentiation of bone marrow MSCs via p53. (a) Representative western blots for the expression of p53. p53 expression was upregulated in bone marrow MSCs following RIPK1 inactivation. Histogram analysis showing the relative protein levels of p53. Data are presented as the means ± SD from three independent experiments (^∗^*p* < 0.05, vs. 0 h group; *n* = 3). (b) The bone marrow MSCs were transfected with sip53 for 24 h; then, the knockdown efficiency of p53 was assessed by western blotting. Representative western blots of the expression of p53. Histogram analysis showing the relative protein levels of p53. Data are presented as the means ± SD from three independent experiments (^∗^*p* < 0.05, vs. siControl; *n* = 3). sip53(#1) showed the highest efficiency among three sip53. (c) The inhibition effect of siPIRK1 on cell proliferation was significantly reversed by sip53, by CCK-8 assay. Values are expressed as the means ± SD from three independent experiments (^∗^*p* < 0.05, vs. siRIPK1; *n* = 3). (d) Alizarin Red staining showed the inhibition of siRIPK1 on osteogenic differentiation was reversed by sip53, and (e) Runx2 expression level in siRIPK1 was increased after transfected with sip53. Values are expressed as the means ± SD from three independent experiments (^∗^*p* < 0.05, vs. siRIPK1; *n* = 3) (scale bar = 200 *μ*m). (f) Oil Red O staining showed the inhibition of siRIPK1 on adipogenic differentiation was reversed by sip53, and (g) PPAR*γ* expression level in siRIPK1 was increased after transfected with sip53. Values are expressed as the means ± SD from three independent experiments (^∗^*p* < 0.05, vs. siRIPK1; *n* = 3) (scale bar = 100 *μ*m). (h) Alcian Blue staining showed the inhibition of siRIPK1 on chondrogenic differentiation was reversed by sip53, and (i) Sox9 expression level in siRIPK1 was increased after transfected with sip53. Values are expressed as the means ± SD from three independent experiments (^∗^*p* < 0.05, vs. siRIPK1; *n* = 3) (scale bar = 100 *μ*m).

To explore the mechanism of how RIPK1 regulates cell biology in bone marrow MSCs, we focused on p53, which is an important regulator for cell fate decision [36–39]. As shown in Figure 4(a), after knockdown of RIPK1 in bone marrow MSCs, the expression of p53 showed a significant increase at all time points. Next, to investigate the role of p53 in RIPK1-deficient bone marrow MSCs, we silenced p53 in RIPK1-deficient bone marrow MSCs. Knockdown efficacy of p53 in bone marrow MSCs was confirmed by western blot analysis (Figure 4(b)). The suppression of p53 could significantly reverse RIPK1 deficiency-induced cell proliferation inhibition (Figure 4(c)). As shown in Figures 4(d) and 4(e), after knockdown of RIPK1 in bone marrow MSCs, the osteogenic differentiation was inhibited, and this effect was reversed by sip53. Meanwhile, RIPK1 deficiency also decreased adipogenic differentiation in bone marrow MSCs which was rescued by sip53 (Figures 4(f) and 4(g)). In addition, we also examined the effect of p53 on chondrogenesis in RIPK1-deficient bone marrow MSCs. The results demonstrated that sip53 rescued the RIPK1 deficiency-induced impairment in chondrogenic differentiation in bone marrow MSCs (Figures 4(h) and 4(i)).

Taken together, p53, at least in part, was involved in RIPK1 deficiency-mediated suppression of proliferation and differentiation in bone marrow MSCs.

### 3.5. p53 Mediates Suppression of Cell Survival in RIPK1-Deficient Bone Marrow MSCs

**Figure 5:**
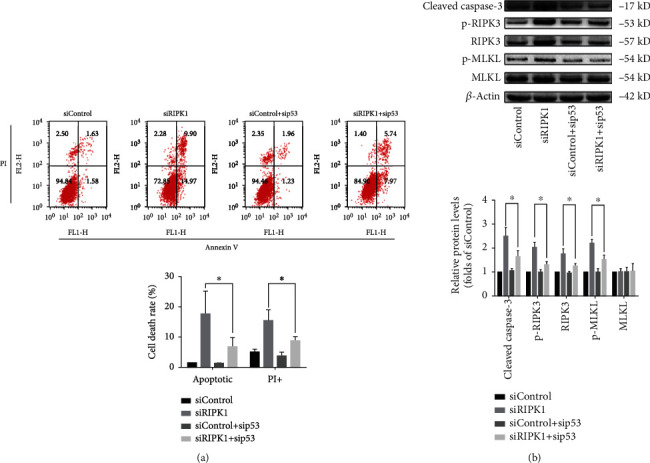
RIPK1 regulated cell survival of bone marrow MSCs via p53. (a) Representative graphs were analyzed using the flow cytometer and quantification analysis suggested that knockdown of p53 significantly reduced the apoptotic and necrotic cell death rate in RIPK1-deficient bone marrow MSCs. Values are expressed as the means ± SD from three independent experiments (^∗^*p* < 0.05, vs. siRIPK1; *n* = 3). (b) Representative western blots for the expression of Cleaved caspase-3, p-RIPK3, p-MLKL, and corresponding total protein. Histogram analysis showing the relative protein levels of Cleaved caspase-3, p-RIPK3, p-MLKL, and corresponding total protein. Data are presented as the means ± SD from three independent experiments (^∗^*p* < 0.05, vs. siRIPK1; *n* = 3).

To further confirm the mechanism of p53 in RIPK1 deficiency-induced cell death, p53 was silenced with sip53(#1). Both apoptotic and necrotic cell rates in response to RIPK1 knockdown were significantly decreased in p53 silenced groups (Figure 5(a)). Furthermore, silence of p53 dramatically protected RIPK1-deficient bone marrow MSCs against apoptosis and necroptosis (Figure 5(a)). The expression of Cleaved caspase-3, p-RIPK3, RIPK3, and p-MLKL increased significantly in RIPK1-deficient bone marrow MSCs, which was reversed by sip53 (Figure 5(b)). The results demonstrated that p53 mediated RIPK1 deficiency-induced cell death in bone marrow MSCs.

### 3.6. p53 Mediates Mitochondrial Homeostasis Disruption in RIPK1-Deficient Bone Marrow MSCs

**Figure 6:**
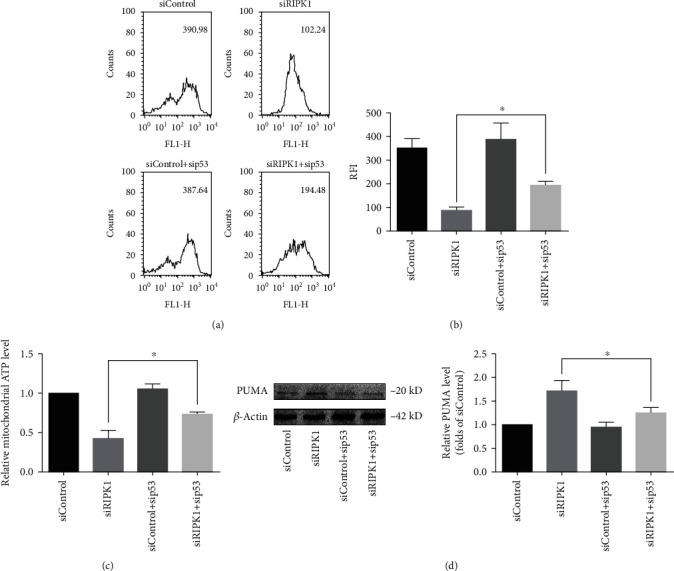
RIPK1 maintained mitochondrial homeostasis of bone marrow MSCs via p53. (a) Representative graphs illustrated that the increase of mPTP opening after knockdown of RIPK1 in bone marrow MSCs was significantly reversed by sip53. (b) The quantitative mPTP in bone marrow MSCs was demonstrated by RFI. Data are expressed as the means ± SD from three independent experiments (^∗^*p* < 0.05, vs. siRIPK1; *n* = 3). (c) The decrease of mitochondrial ATP production after knockdown of RIPK1 in bone marrow MSCs was significantly reversed by sip53. Values are expressed as the means ± SD from three independent experiments (^∗^*p* < 0.05, vs. siRIPK1; *n* = 3). (d) Representative western blots for the expression of PUMA. Histogram analysis showing the relative protein levels of PUMA. Data are presented as the means ± SD from three independent experiments (^∗^*p* < 0.05, vs. siRIPK1; *n* = 3).

Mitochondria have been demonstrated as key modulators of the functions of bone marrow MSCs [40]. To this end, we investigate whether p53 is also implicated in mitochondrial homeostasis disruption in RIPK1-deficient bone marrow MSCs. First, the opening rate of mPTP was evaluated in bone marrow MSCs. Compared with the control group, knockdown of RIPK1 promoted the opening of mPTP, and this effect was reversed by sip53 (Figures 6(a) and 6(b)). Meanwhile, sip53 alleviated RIPK1 deficiency-induced mitochondrial ATP depletion (Figure 6(c)).

PUMA is one of the most transcriptional targets of p53 and is required for p53-mediated mitochondria damage [30]. PUMA could serve as a distinct protein to induce the opening of mPTP. In the present study, we showed that PUMA was activated in RIPK1-deficient bone marrow MSCs. Interestingly, p53 deletion in RIPK1-deficient bone marrow MSCs repressed PUMA expression and the opening of mPTP (Figures 6(a) and 6(d)). This finding suggested that p53 mediated mitochondrial homeostasis disruption in RIPK1-deficient bone marrow MSCs.

## 4. Discussion

Bone marrow MSCs play a critical role in the regulation of bone homoeostasis and hematopoiesis [1, 2]. MSCs are multipotent stem cells in the bone marrow, which exhibit self-renewal and multipotency capabilities and serve as the stem cell niche for tissue repair and regeneration [3]. The fates of MSCs in the bone marrow were tightly regulated by various genetic factors. MSC deficiency or dysfunction in the bone marrow could directly contribute to numerous bone diseases [41, 42]. RIPK1 has been identified as a master modulator of the cellular fate decision in tissue homeostasis and development [43]. However, it is unknown whether RIPK1 is required to maintain stem cell properties in bone marrow MSCs.

In our study, we first provided evidence demonstrating that RIPK1 is required for bone marrow MSC survival. Knockdown of RIPK1 in bone marrow MSCs suppressed proliferation and differentiation, while it enhanced apoptosis and RIPK3-mediated necroptosis in vitro. More importantly, we also found that mitochondria were a critical target for RIPK1 and knockdown of RIPK1 led to the opening of mPTP, resulting in the loss of MMP and mtDNA integrity. Furthermore, our findings identified that RIPK1 acts as a novel molecular switch for MSC fate decisions through the p53-PUMA axis. Notably, RIPK1 is a critical mediator for bone marrow MSC survival.

The impact of RIPK1 for the cell fate decision has been controversially discussed. On the one hand, multiple sources of evidence have indicated that RIPK1 could initiate RIPK3/MLKL-dependent necroptosis or caspase-dependent apoptosis by its kinase domain [43–46]. On the other hand, deletion of RIPK1 could sensitize cells to apoptosis or necroptosis through impaired MAPK pathway activation [16]. In addition, in human patients with inherited RIPK1 deficiency, defective differentiation of hematopoietic progenitors in the bone marrow could result in immune dysfunctions and/or system inflammation [14]. These findings reveal that the requirement of RIPK1 for the cell fate decision may depend on the cellular context and local tissue microenvironment. Considering the difficulty of distinguishing the cell-intrinsic effects from the systemic effects of RIPK1 deficiency in vivo, we generated RIPK1-deficient bone marrow MSCs by genetic manipulations. Knockdown of RIPK1 in bone marrow MSCs could inhibit cell proliferation, differentiation, and migration and promote cell death in vitro. Furthermore, we found that the characteristics of bone marrow MSC cell death after knockdown of RIPK1 appear to be associated with caspase-dependent apoptosis and RIPK3/MLKL-dependent necroptosis. This result is in agreement with previous studies in other types of cells, suggesting that the kinase-independent or scaffold-dependent functions of RIPK1 are indispensable for maintaining bone marrow MSC survival.

Extensive studies have shown that mitochondria are essential for cell survival in bone marrow MSCs [26, 40]. These data pointed mitochondrial homoeostasis as the convergent point for many programmed cell death pathways [47]. The opening of mPTP seems to be a critical step for apoptosis and necroptosis [47]. In apoptosis, the opening of mPTP leads to the collapse of MMP and the release of mitochondrial contents such as cytochrome c and then triggers caspase cascade and subsequently activates apoptosis [23]. During necroptosis, PGAM5-mediated mPTP opening facilitates the phosphorylation of RIPK3 and MLKL and then translocates to the plasma membranes and thus disrupts cell [23]. In addition, the opening of mPTP promotes the cytosolic release of mtDNA and depletion of mitochondrial ATP [30]. Under RIPK1 deficiency condition, ZBP1, as a DNA sensor, could be activated by cytosolic mtDNA and trigger RIPK3-dependent necroptosis [30, 47, 48]. In our study, we observed that knockdown of RIPK1 stimulated the opening of mPTP, disrupted mitochondrial homeostasis and MMP, and thus promoted the release of mtDNA and cytochrome c from mitochondria. However, details of how mitochondria mediated apoptosis/necroptosis initiation are still unclear.

The p53-PUMA axis has been reported as a key molecular switch for stem cell fate determination [49]. In response to stress, the hyperactivation of p53 induced cell death via triggering PUMA, a BH3-only proapoptotic protein in hematopoietic stem cells and intestinal stem cells [50–52]. Previous studies have also found that deletion of PUMA protected cell by diminishing apoptosis and necroptosis [53, 54]. However, little is known about the mechanism of p53-PUMA axis in bone marrow MSC survival. Our results indicated that knockdown of RIPK1 promoted the expression of p53 and PUMA, suggesting that the p53-PUMA axis was activated in RIPK1-deficient bone marrow MSCs. It has been demonstrated that PUMA could directly regulate mitochondrial homoeostasis in bone marrow MSCs. Furthermore, we found that knockdown of p53 suppressed the activation of PUMA and mitochondrial dysfunction and then protected against RIPK1 deficiency-induced apoptosis and necroptosis in bone marrow MSCs.

In our study, several limitations need to be pointed out. First, we chose primary bone marrow MSCs to explore the biochemical function of RIPK1 in vitro, and the conclusions were not simply equal to what happens in vivo. However, considering the difficulty of distinguishing the cell-intrinsic effects from the systemic effects of RIPK1 deficiency in vivo, there are advantages to using primary cell models, in which culture environments can be controlled more precisely. Second, it is difficult for us to access to healthy human bone marrow MSCs. Thus, SD rats bone marrow MSCs were used as alternative in our experiment. Emerging evidence has reported that the molecular function of RIPK1 might be diverse in different species [14]. In the future, our studies using human bone marrow MSCs are warranted. Third, our data only elaborate one aspect of RIPK1 function in bone marrow MSCs. In fact, various molecules may contribute to RIPK1-mediated mitochondrial homeostasis and cell fate decision in bone marrow MSCs. Therefore, further experiments are needed to explore more factors involved in RIPK1-mediated mitochondrial homeostasis and cell fate decision.

**Figure 7:**
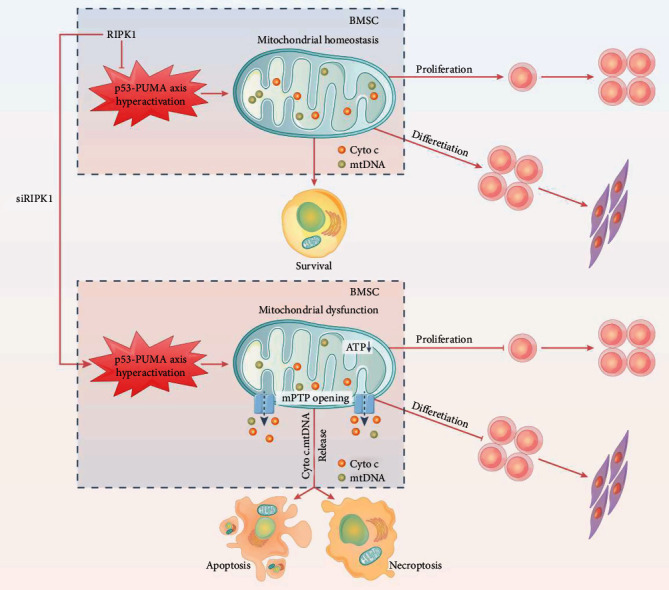
Schematic illustration showing that p53-PUMA-mediated mPTP is involved in RIPK1 deficiency-induced apoptosis and necroptosis of bone marrow MSCs.

In summary, we first explored the function and mechanisms of RIPK1 on bone marrow MSCs and summarized in Figure 7. We found that RIPK1 deficiency induced apoptosis and necroptosis in bone marrow MSCs, which is linked to mitochondrial dysfunction. Furthermore, we identified that the p53-PUMA axis pathway was involved in mitochondrial dysfunction in RIPK1-deficient bone marrow MSCs. Our findings highlighted that RIPK1 was indispensable for bone marrow MSC survival.

## Data Availability

The data used to support the findings of this study are available from the corresponding author upon request.

## Abbreviations

ONFH: Osteonecrosis of the femoral head
RIPK1: Receptor-interacting protein kinase 1
RIPK3: Receptor-interacting protein kinase 3
MLKL: Mixed lineage kinase domain-like
HSCs: Hematopoietic stem cells
MSCs: Mesenchymal stem cells
MDS: Myelodysplastic syndrome
MM: Multiple myeloma
PUMA: p53 upregulated modulator of apoptosis
mPTP: Mitochondrial permeability transition pore
mtDNA: Mitochondrial DNA
MMP: Mitochondrial membrane potential
siRNA: Small interfering RNA.
